# The Essential Role of H19 Contributing to Cisplatin Resistance by Regulating Glutathione Metabolism in High-Grade Serous Ovarian Cancer

**DOI:** 10.1038/srep26093

**Published:** 2016-05-19

**Authors:** Zhi-Guo Zheng, Hong Xu, Sha-Sha Suo, Xiao-Li Xu, Mao-Wei Ni, Lin-Hui Gu, Wei Chen, Liang-Yan Wang, Ye Zhao, Bing Tian, Yue-Jin Hua

**Affiliations:** 1 Institute of Nuclear-Agricultural Sciences, Zhejiang University, 310029, Hangzhou, China; 2 Institute of Zhejiang Cancer Research, Zhejiang Cancer Hospital, Hangzhou, 310022, China

## Abstract

Primary and acquired drug resistance is one of the main obstacles encountered in high-grade serous ovarian cancer (HGSC) chemotherapy. Cisplatin induces DNA damage through cross-linking and long integrated non-coding RNAs (lincRNAs) play an important role in chemical induced DNA-damage response, which suggests that lincRNAs may be also associated with cisplatin resistance. However, the mechanism of long integrated non-coding RNAs (lincRNAs) acting on cisplatin resistance is not well understood. Here, we showed that expression of lin-RECK-3, H19, LUCAT1, LINC00961, and linc-CARS2-2 was enhanced in cisplatin-resistant A2780-DR cells, while transcriptome sequencing showed decreased Linc-TNFRSF19-1 and LINC00515 expression. Additionally, we verified that different H19 expression levels in HGSC tissues showed strong correlation with cancer recurrence. H19 knockdown in A2780-DR cells resulted in recovery of cisplatin sensitivity *in vitro* and *in vivo*. Quantitative proteomics analysis indicated that six NRF2-targeted proteins, including NQO1, GSR, G6PD, GCLC, GCLM and GSTP1 involved in the glutathione metabolism pathway, were reduced in H19-knockdown cells. Furthermore, H19-knockdown cells were markedly more sensitive to hydrogen-peroxide treatment and exhibited lower glutathione levels. Our results reveal a previously unknown link between H19 and glutathione metabolism in the regulation of cancer-drug resistance.

Epithelial ovarian cancer mortality ranks the highest in gynecological cancer, and overall survival rates have changed little in the last 30 years^1^,^2^. Most patients with epithelial ovarian cancer have high-grade serous cancer (HGSC), with 75% of patients presenting an advanced stage^3^. Platinum-based chemotherapy (in combination with paclitaxel or docetaxel) is first-line chemotherapy for advanced disease^4^. The main limitation to the clinical usefulness of platinum (cisplatin or carboplatin) is the high incidence of chemoresistance. Most cisplatin-resistant patients also fail to respond to carboplatin^5^.

In order to overcome platinum resistance, a deeper understanding of molecular mechanisms is necessary. Existing experimental results show that multiple factors contribute to cisplatin resistance in ovarian cancer, including reduced intracellular drug concentration, activated cisplatin inactivation by antioxidant systems, increased DNA-repair capacity, and defects in signaling pathways^5^. Cellular cisplatin accumulation is mainly determined by copper transporter-1, responsible for cisplatin uptake^6^,^7^, and *ABCB1*, encoding multidrug-resistant protein 1 (MDR1) involved in increasing platinum-drug export^8^,^9^. Recent research showed that MDR1 overexpression through promoter fusion contributed to acquired-resistance in HGSC^10^. Activated cisplatin is susceptible to inactivation by antioxidant systems, and glutathione (GSH) is one of the most important antioxidants. Most cisplatin-resistant cancer cells are associated with increased GSH synthesis^11^,^12^ and formation of GSH-cisplatin conjugates mediated by glutathione S-transferase P1 (GSTP1) is responsible for cisplatin inactivation^5^. The majority of cisplatin-induced DNA lesions are removed through homologous recombination, nucleotide-excision repair, and mismatch-repair pathways, which are up-regulated in drug-resistant cancer cells^13^. Furthermore, reversion of germline mutations in *BRCA1* or *BRCA2* and loss of *BRCA1*-promoter methylation enhanced acquired resistance to platinum-based chemotherapy^10^. Defects in other signaling pathways have also been found in cisplatin-resistant ovarian cancer. For example, elevated levels of the anti-apoptotic protein BCL-XL correlates with cisplatin resistance and tumor recurrence in ovarian cancer^15^. Also, ovarian cancer cells upregulating components of the autophagy pathway have been shown to acquire cisplatin resistance^16^.

An increasing number of studies have found that long integrated non-coding RNAs (lincRNAs) play an important role in DNA-damage response^17^. For example, lincRNA-p21 induced by p53 is responsible for repressing p53-dependent genes that interfere with apoptosis. Furthermore, lincRNA-p21 acts as an inhibitor, interacting with ribonucleoprotein K, which is then recruited to gene-promoter regions^18^. A prostate-cancer outlier lincRNA (PCAT-1) is involved in genotoxic stress response. Expression of PCAT-1 increases cell sensitivity to small-molecule PARP1 inhibitors by repressing BRCA2^19^. Platinum induces DNA damage through cross-linking, which suggests that lincRNAs may be associated with cisplatin resistance. LincRNA urothelial cancer-associated 1 (UCA1) is highly expressed in cisplatin-resistant bladder cancer and ovarian cancer cells. Overexpression of UCA1 in SKOV3 cells significantly increases expression of SRPK1 and anti-apoptosis proteins^20^,^21^. However, the mechanism involved in lincRNA association with cisplatin resistance needs further study.

LincRNA H19 is involved in tumor development, progression and metastasis. H19 was initially proposed as a tumor-suppressor^22^, however, H19 is abundantly expressed in a majority of human cancers, suggesting that H19 may play a role in oncogenesis^23^,^24^,^25^. Recent analysis showed that H19 expression was associated with cancer metastasis. Several conflicting studies linked H19 to cancer metastasis, with three reports revealing that H19 overexpression increased bladder cancer, pancreatic ductal adenocarcinoma, and gastric-cancer metastasis^26^,^27^,^28^. Another study showed that H19 contributed to metastatic suppression in hepatocellular carcinoma^29^. H19 was also found to be overexpressed in drug-resistant cell lines, such as doxorubicin-resistant breast- and liver cancer cells^30^,^31^ and a cisplatin-resistant ovarian cancer cell line^32^.

Despite efforts, cisplatin-resistance mechanisms in HGSC are not well understood. In this study, we employed transcriptome sequencing to systematically identify the differentially expressed lincRNAs in cisplatin sensitive (A2780) and resistant (A2780-DR) cells. Selected lincRNAs were confirmed by fluorescence quantitative real-time PCR (qRT-PCR) in cells and tissues. We also performed lentivirus-mediated RNA interference (RNAi) silencing and quantitative proteomics analysis to explore the functions of lincRNA H19 in cisplatin resistance, to elucidate how this lincRNA participates in a variety of cellular processes, and enhance our understanding of the mechanisms of HGSC drug-resistance. Our results indicate a previously unknown link between H19, oxidative reduction, and GSH metabolism in the regulation of cancer-drug resistance.

## Results

### Transcriptome analysis of A2780 and A2780-DR cells

**Figure 1:**
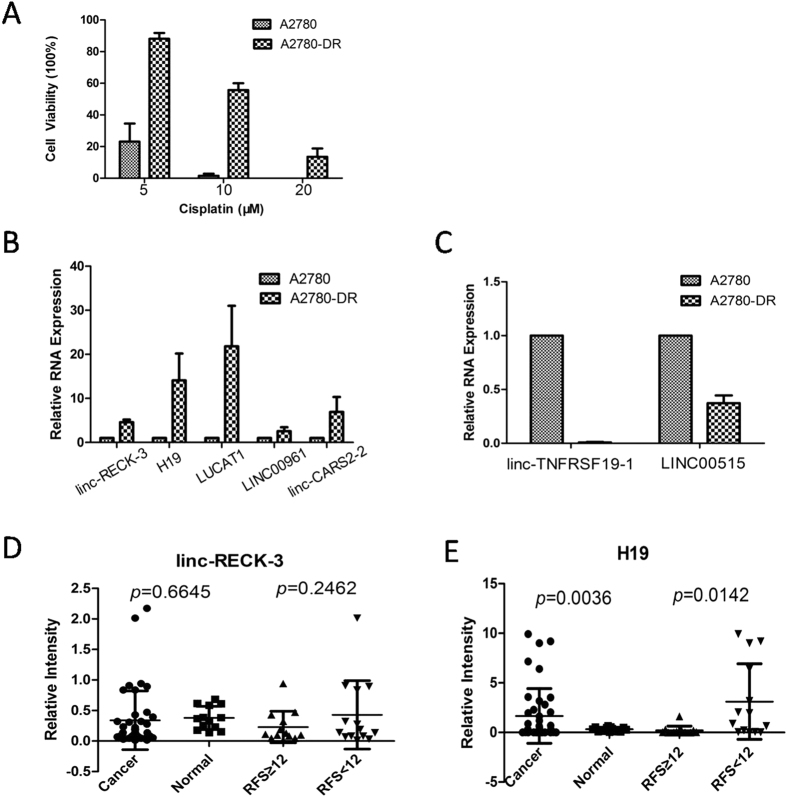
LincRNAs associated with cisplatin resistance. Toxicity analysis of cisplatin sensitive. (A2780) and resistant cells (A2780-DR) under cisplatin treatment (5, 10, 20 uM) in 24 hours; (**B**) Linc-RECK-3, H19, LUCAT1, LINC00961 and linc-CARS2-2 expression level in cisplatin sensitive and resistant cells; (**C**) Linc-TNFRSF19-1 and LINC00515 expression level in cisplatin sensitive and resistant cells; (**D**) Linc-RECK-3 expression level in different tissues and in different RFS patients samples; (**E**) H19 expression level in different tissues and different RFS patients samples.The statistic analysis of (**D**,**E**) was performed by t-test, two sided, equal variances not assumed.

Our previous work demonstrated that A2780-DR cells are cisplatin resistance^33^. The resistance of A2780-DR to cisplatin was significantly increased according to clonogenic assay (Fig. 1A). When cells were treated with 10 μM cisplatin for 24 hours, the percentage of viable clones was 1.5% and 55.7% for A2780 and A2780-DR cells, respectively. A2780-DR cells showed a uniquely stable cisplatin-resistant phenotype for at least 8 months in drug-free complete medium.

In order to understand the mechanisms involving lincRNA in cisplatin resistance, transcriptome sequencing without ribosomal RNA was undertaken. All RNA-seq data were aligned to hg19 using TopHat/Bowtie^34^ with default parameters. Readings that uniquely mapped to the genome were 34401987 and 21750340 for A2780 and A2780-DR cells, respectively (S1). Differentially expressed lincRNAs between A2780 and A2780-DR cells were obtained by analysis using two databases. There were 296 differentially expressed lincRNAs between A2780 and A2780-DR cell lines based on the LNCipedia database^35^, of which 183 lincRNAs were downregulated and 113 were upregulated in drug resistant cells (S2). Based on the Ensembl database^36^, 140 lincRNAs were found to be differentially expressed, with 82 lincRNAs exhibiting low expression levels and 58 lincRNAs exhibiting high expression levels in cisplatin resistant cells (S3).

10 lincRNAs were selected for qRT-PCR validation, of which seven were highly expressed and three exhibited low expression levels in drug-resistant A2780-DR cells based on the Integrative Genomics Viewer tool (http://www.broadinstitute.org/igv/) (Supplementary Figures 1 and 2). Lin-RECK-3, H19, LUCAT1, LINC00961, and linc-CARS2-2 showed significantly increased expression levels in A2780-DR cells, which changed by ~4.5-, ~14.09-, ~21.8-, ~2.5-, and ~6.9-fold, respectively (Fig. 1B). Linc-TNFRSF19-1 and LINC00515 showed significantly decreased expression levels in cisplatin-resistant cells (Fig. 1C).

To validate the relationship of those lincRNAs putatively involved in cisplatin resistance, lin-RECK-3 and H19 were chosen as candidates for further testing. 41 cases of HGSC tissues and 13 cases of normal ovarian epithelium from benign-tumor patients were selected for verification. To better understand the relationship with cancer recurrence, 27 cases of patients with recurrence after hospital treatment were selected, with the recurrence free survival (RFS) of 12 months established as the boundary for the two groups (13 patients had an RFS ≥12 months). There was no significant difference in linc-RECK-3 expression between cancer and normal tissues (*p* = 0.6645) and no relationship with RFS (*p* = 0.2462; Fig. 1D). However, H19 was highly expressed in cancer tissues (*p* = 0.0036) and showed a significant relationship with RFS (*p* = 0.0142; Fig. 1E), indicating that HGSC patients with higher H19 expression levels had higher risk of ovarian-cancer recurrence.

### H19 overexpression contributes to cisplatin resistance

**Figure 2:**
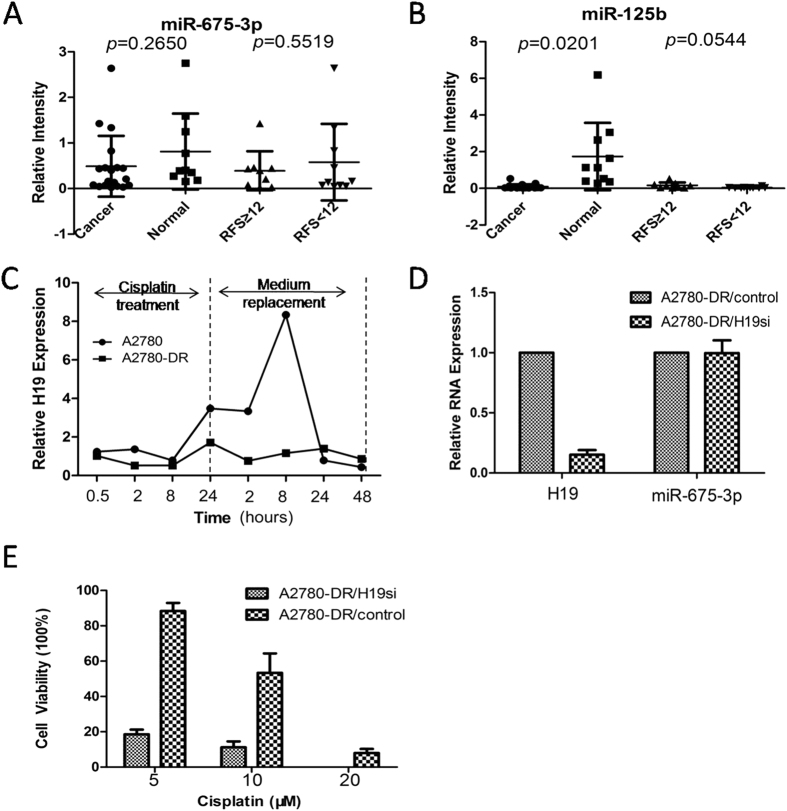
H19 over-expression contributes to cisplatin resistance. (**A**) miR-675-3p expression level in different tissue and different RFS patients samples; (**B**) miR-125b expression level in different tissue and different RFS patients samples; The statistic analysis of (**A,B**) was performed by t-test, two sided, equal variances not assumed. (**C**) Dynamic changes of H19 expression level in response to cisplatin treatment. The 1–8 time course successively present 0.5, 2, 8, 24 hours after cisplatin treatment and 2, 8, 24, 48 hours after medium replacement. (**D**) H19 and miR-675-3p expression level after H19 siRNA interference. **(E)** Toxicity sensitivity analysis of A2780-DR/H19si and A2780-DR/control cells under cisplatin treatment in 24 hours.

H19 function can be changed by sequence mutation, encoding microRNAs, transcript expression, and other factors. We obtained the sequence of full-length H19, including five exons and four introns. There was no difference in the H19 sequence between drug-resistant and -sensitive cell lines. Although H19 can function as a miR-675-3p precursor, there was no significant difference in miR-675-3p levels between 19 HGSC and 10 normal ovarian epithelia from benign ovarian tumors (*p* = 0.2650; Fig. 2A). The expression of miR-675-3p was also unrelated to RFS (*p* = 0.5519; Fig. 2A). As a positive control, miR-125b was highly expressed in normal tissues (*p* = 0.0201; Fig. 2B). H19 was induced by cisplatin treatment in drug-sensitive cells, but not in drug-resistant cell lines. H19 expression increased significantly after 24 hours of drug treatment in the drug-sensitive cell line. H19 expression reached its peak 8 hours after replacement of the culture medium (Fig. 2C), indicating that H19 is involved in the response to cisplatin treatment.

To further prove the importance of H19 in the process of cisplatin resistance, small hairpin RNA (shRNA) transfection was performed using three sequences designed to target H19. The second sequence had the highest interference efficiency (Supplementary Figure 3). The H19-interference cell line (A2780-DR/H19si) was established using lentivirus transfection and puromycin selection with the second sequence. After interference, H19 expression levels decreased nearly 6-fold, but miR-675-3p expression was unaffected (Fig. 2D). The sensitivity of the A2780-DR/H19si cell line to cisplatin increased significantly, with the survival rate decreased to 18.55%, 11.20%, and 0% following treatment with 5, 10, and 20 μmol cisplatin. These survival rates were lower than the rates of the A2780-DR/control cell line following same treatments (88.36%, 53.35%, and 7.95%, respectively; Fig. 2E). Therefore, these results suggest that H19 overexpression contributes to cisplatin resistance, rather than the mutated H19 variant or its encoding microRNA.

**Figure 3:**
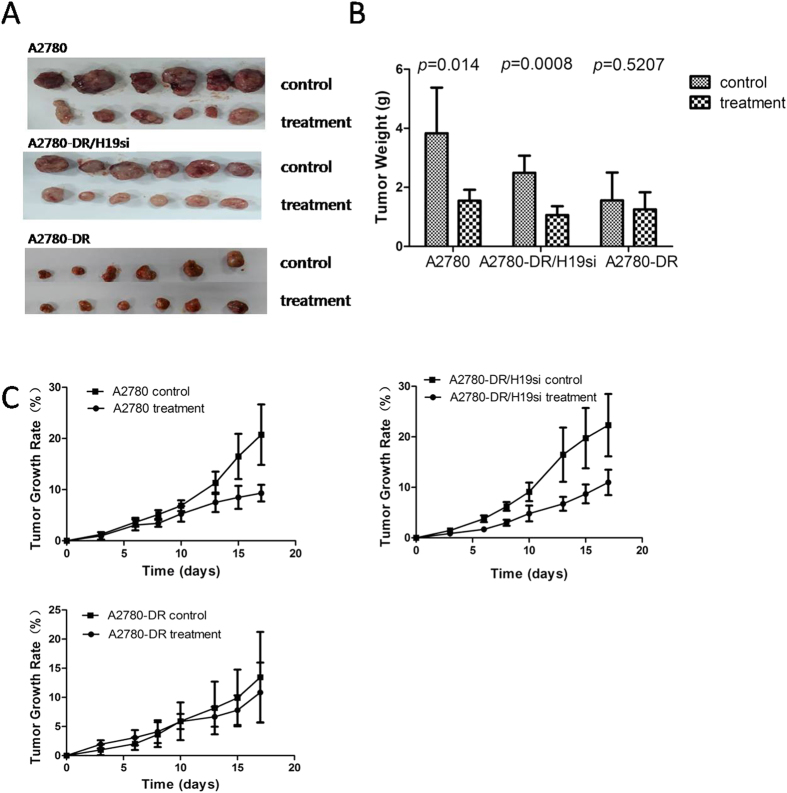
Tumor formation comparison of A2780, A2780-DR/H19si, A2780-DR after cisplatin treatment. (**A**) Tumor size between control and cisplatin treatment group of three cell lines. Samples were collected after the mice were sacrificed. (**B**) Tumor weight between two sub-groups (control and treatment) of three cell lines. Tumor weight was measured after the mouse was sacrificed. T-test was performed in this experiment, equal variances not assumed. (**C**) Tumor growth rates between control and cisplatin treatment group of three cell lines. Tumor formation was assessed three times a week for three weeks with cisplatin injection per week. Tumor volume was calculated based on v = ½ab^2^; growth rate was calculated based on 
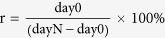
, N equals the day the tumor size was measured.

To further test this hypothesis, three mouse lines injected with A2780, A2780-DR, and A2780-DR/H19si were established. After three injections of cisplatin, the size of tumors formed by A2780 and A2780-DR/H19si cells dramatically decreased compared to control tumors (Fig. 3A). The tumor weight and volume decreased by more than 50% [*p* = 0.014 and *p* = 0.0008, respectively; (Fig. 3B,C)]. However, the tumor initiated with A2780-DR cells showed no observable response to cisplatin treatment (Fig. 3A–C). The mouse model results support an important role for H19 in development of cisplatin resistance.

### H19 contributes to cisplatin resistance by promoting GSH metabolism

**Figure 4:**
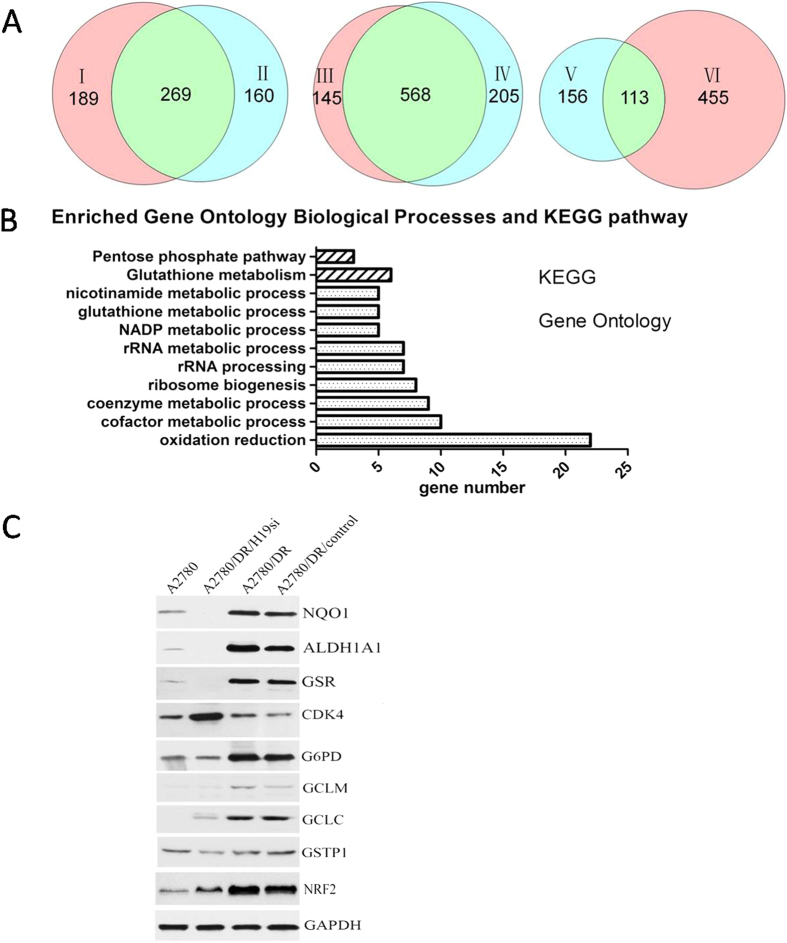
Proteomic analysis and comparison among different cell lines. Proteomic analysis of A2780, A2780-DR, A2780-DR/H19si and A2780-DR/control cells, the common and the difference. І presented the different expression proteins between A2780-DR and A2780 cells, ІІ presented the different expression proteins between A2780-DR/control and A2780 cells, III presented the different expression proteins between A2780-DR and A2780-DR/H19si cells, IV presented the different expression proteins between A2780-DR/H19si and A2780-DR/control cells. V presented the common difference by I and II comparison, VI presented the common difference by III and IV comparison; (**B**) KEGG and Gene Ontology analysis; (**C**) Western blot validation of different expression proteins (NQO1, ALDH1A1, GSR, CDK4, G6PD, GCLC, GCLM, GSTP1, NRF2).

To demonstrate how H19 contributes to cisplatin-resistance in ovarian cancer, proteomic analysis was carried out on A2780, A2780-DR, A2780-DR/H19si, and A2780-DR/control cells. Proteins expression levels were identified and quantified using MaxQuant software (http://medusa.biochem.mpg.de/maxquant_doku/). The experiments were repeated twice and the false-positive rate was set to be <1%. Based on label-free quantification intensity ratios (>1.67 or <0.6) in proteins that have two or more unique peptides, 458 proteins were found to be differentially expressed between A2780-DR and A2780 cells, of which 213 proteins were downregulated and 245 upregulated in A2780-DR (S4). There were 429 differentially expressed proteins found between A2780-DR/control and A2780 cells, of which 215 proteins were downregulated and 214 upregulated in the A2780-DR/control (S5). Furthermore, A2780-DR and A2780-DR/control shared 269 differentially expressed proteins, of which 134 were downregulated and 135 were upregulated (Figs 4A, S6). There were 713 differentially expressed proteins found between A2780-DR and A2780-DR/H19si cells, of which 349 proteins were downregulated and 364 were upregulated in A2780-DRcells (S7). There were 773 differentially expressed proteins found between A2780-DR/control and A2780-DR/H19si cells, of which 413 proteins were downregulated and 360 were upregulated in A2780-DRcells (S8). Furthermore, A2780-DR and A2780-DR/control shared 568 differentially expressed proteins, of which 290 proteins were downregulated and 278 were upregulated (Figs 4A, S9). There were 113 differentially expressed proteins found between A2780-DR, A2780-DR/control and A2780, A2780-DR/H19si cells, of which 42 proteins were downregulated and 71 upregulated in A2780-DR (Figs 4A, S10).

In order to understand the biological relevance of the identified proteins, the DAVID bioinformatics platform (http://david.abcc.ncifcrf.gov/) was used to categorize the differentially expressed proteins. This categorization includes gene ontology (GO) and Kyoto Encyclopedia of Genes and Genomes (KEGG) pathway determination (Fig. 4B). 113 differentially expressed proteins were classified into several significant groups of biological processes, including oxidation reduction (22/113; 19.5%), NADP metabolic processes (5/113; 4.4%), cofactor metabolic processes (10/113; 8.8%), coenzyme metabolic processes (9/113; 8%), ribosome biogenesis (8/113; 7.1%), and glutathione metabolic processes [5/113; 4.4%; (S11)]. At the same time, proteins were classified into several KEGG pathways, including glutathione metabolism (6/113; 5.3%), pentose phosphate pathway (3/113; 2.7%), and pyruvate metabolism [3/113; 2.7%; (S12)].

**Figure 5:**
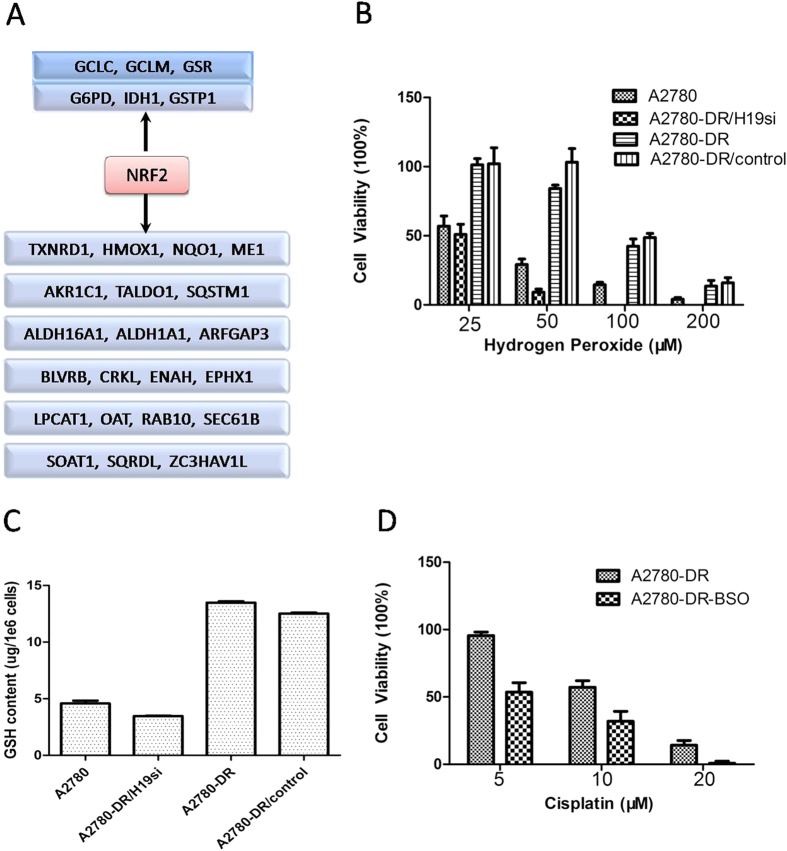
H19 plays an important role in cisplatin resistance by regulating GSH metabolism. (**A**) “NRF2-targeted” genes identified by proteomic analysis and nrf2ome database (http://nrf2.elte.hu/). (**B**) Cell viability of four cell lines under Hydrogen peroxide treatment (25,50,100,200 μM) in 24 hours. (**C**) Intracellular GSH level of four cell lines, the GSH content were determined by the HPLC method. (**D**) Cisplatin sensitivity of A2780-DR cell after BSO treatment. Cisplatin resistant cell A2780-DR treated with BSO (100 μM) for 24 hours. After medium replacement, two groups of cells (A2780-DR, A2780-DR-BSO) were treated with cisplatin (5 μM, 10 μM, 20 μM) for next 24 hours and the cell viability was measured after that.

22 differentially expressed proteins participated in oxidation reduction processes (S13). Among these, NQO1 and ALDH1A1 were confirmed by Western blot analysis (Fig. 4C). There were 27 NRF2-targeted proteins identified by proteomic analysis and the nrf2ome database (http://nrf2.elte.hu/) (Figs 5A, S14). Six NRF2-targeted proteins were involved in the GSH-metabolism pathway, including the GSH-production enzymes (GCLM and GCLC), regeneration enzymes (G6PD, GSR, and IDH1), and utilization enzyme GSTP1 (Supplementary Figures 4–5, S15). These proteins were observed at higher concentrations in the cisplatin-resistant cell lines. Among these proteins, the expression level of GSR, G6PD, GCLC, GCLM, GSTP1 with NRF2 in different cell lines were confirmed by Western blot analysis (Fig. 4C). Besides the level of GSTP1 wasn’t change very obvious in those four cell lines, all of other proteins’ level was raised substantially in cisplatin resistant cells. To further verify whether NRF2 level would contributes to cisplatin resistance, RNA interference assay was performed. A2780-DR cells with NRF2 knockdown were more sensitive to cisplatin treatment compared to cells transfected with empty vector (Supplementary Figure 6), which supported our hypothesis that NRF2 level was related to cisplatin sensivity in our cell lines and may be regulated through H19-dependent pathway.

To test if the cells have different responses under oxidative stress conditions, survival rates following hydrogen peroxide (H_2_O_2_) treatment were measured. The A2780 and A2780-DR/H19si cell lines were more sensitive to H_2_O_2_ than the cisplatin-resistant cell lines [A2780-DR and A2780-DR/control; (Fig. 5B)]. Only 4.07% of the A2780 cells survived and there were no surviving A2780-DR/H19si cells after 200 μmol H_2_O_2_ treatment. Both A2780-DR and A2780-DR/control cells displayed survival rates of ~15% under the same conditions. To further demonstrate the correlation between H19 expression and GSH metabolism, the GSH content was measured in parallel. The levels of GSH in the cisplatin-resistant cell lines were higher than the cisplatin-sensitive cell lines (Fig. 5C), indicating that H19 knockdown in A2780-DR results in decreased GSH activity, which contribute to cisplatin-resistance through activated cisplatin inactivation and anti-oxidative effect. To test if GSH levels do contribute to cisplatin resistant in our cell lines, we treated A2780-DR cells with buthionine sulfoximine (BSO) which was a GSH synthase inhibitor and cell viability was measured under different doses of cisplatin. After the GSH level was decreased in A2780-DR cells about 70% (Supplementary Figure 7), the cell became more sensitive to cisplatin compared to non-treated ones (Fig. 5D), indicating the GSH in A2780 cells had a positive relativity with its cisplatin resistance.

## Discussion

LincRNAs have emerged as essential molecules in cancer biology and may play an important role in cisplatin resistance. To discover the overall changes in lincRNA levels between cisplatin-sensitive and cisplatin -resistant ovarian cancer cells, we employed transcriptome sequencing and found ~436 candidates, with many of them first reported in this study. Although lincRNA UCA1 was reported an important role in cisplatin resistance^20^,^21^, there was no significant difference observed of UCA1 in the comparison of drug-resistant and -sensitive cell lines in our results (data not shown), suggested that the differential expression of lincRNAs associated with cisplatin resistance had a different mechanism in our cell lines. H19 was found being upregulated in cisplatin-resistant ovarian cancer cell lines^32^, which was confirmed by our study.

H19 function can be changed by sequence mutation, encoding microRNAs, or transcript expression. One potentially functional H19 single-nucleotide polymorphism (rs217727 C >T) was significantly associated with increased risk of gastric cancer in the Chinese Han population^37^. Verhaegh *et al.* reported that an H19 genetic variant (rs2839698 TC genotype) was associated with a decreased risk of bladder cancer in European Caucasians^38^. We found no difference in the H19 sequence in our cell model, suggesting that the mechanism of cisplatin resistance was unrelated to H19 mutation. In addition, H19 can function as miR-675 precursor^39^. MiR-675 is upregulated in human colorectal cancer, where it regulates cancer development through downregulation of its target RB gene^40^. H19 may cause genetic restriction of the placenta before birth by regulated processing of miR-675, which suppresses growth and Igf1r expression^41^. In this study, miR-675-3p expression was not associated with RFS in ovarian cancer patients, and the expression of miR-675-3p was not affected by H19 knockdown, indicating that H19 involvement in cisplatin resistance is not related to miR-675-3p. H19 was induced by cisplatin treatment in drug-sensitive cells, but not in drug-resistant cell lines. After H19 interference, H19 expression significantly decreased, and the sensitivity to cisplatin increased significantly. In summary, the mechanism of H19 involvement in cisplatin resistance is related to the overexpression of H19 transcription.

**Figure 6:**
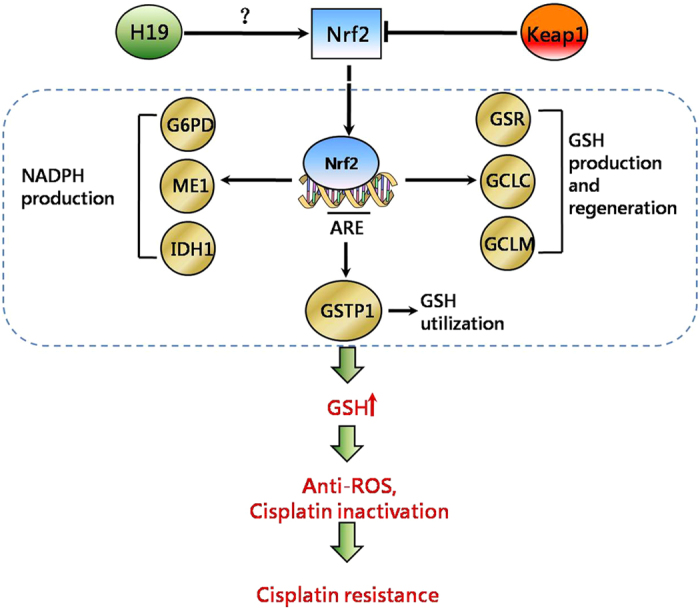
Hypothesis model of how H19 contributes to cisplatin resistance in ovarian cancer cells. Nrf2 is degrated by Keap1 in drug sensitive cells and stabled by H19 in drug resistant cells. “Nrf2-targeted” genes which involved in GSH metabolism transcription and GSH increase in cisplatin resistant cells. High GSH causes cisplatin inactivation by GSTP1 and reduces free radicals, resulting in cisplatin resistance.

To better resolve the specific mechanism involved in H19 regulation of gene expression or protein translation, we looked for potential H19 regulating proteins. A label-free quantitative proteomic method was performed with bioinformatic analysis undertaken using the DAVID platform. We found that H19 mainly regulates oxidative stress and cell-cycle genes, and the primary route of cisplatin resistance involved oxidative-stress pathways, especially NRF2-targeted genes in the GSH pathway (Fig. 6). This is, to the best of our knowledge, the first link of the H19 gene with the GSH pathway contributing to cisplatin resistance. Previous studies reported that increased cellular GSH levels were correlated with cisplatin resistance^11^,^12^,^42^,^43^, and GSH depletion by buthionine-sulfoximine increased sensitivity to cisplatin^44^,^45^. These results suggested that intracellular GSH levels play an important role in cisplatin resistance. GSH production enzymes (GCLM and GCLC) and regeneration enzymes (G6PD and GSR) were found at higher concentrations in cisplatin-resistant cell lines^46^,^47^,^48^,^49^,^50^ which were also confirmed in our study. Furthermore, H19 regulates proteins, such as GSR, G6PD, GCLC, GCLM, GSTP1 and NQO1, which all are NRF2-target genes^51^. NRF2 is an important regulator of the expression of antioxidant molecules within the cell^52^. Therefore, H19 may play an important role in the antioxidant defense through participation with NRF2 pathway. Further research is needed regarding the role of H19 with transcription factors regulating the redox pathway.

Additionally, H19 is involved in tumor development, progression, metastasis, and drug resistance. Disease-free survival from the first biopsy to the first episode of recurrence was significantly shorter in bladder carcinoma patients with tumors having more H19-positive cells^53^. We have found that H19 is highly expressed in ovarian cancer patients that have short RFS. The expression of H19 in an individual biopsy may be considered a predictive tumor marker for selecting those patients who would benefit from this form of treatment. However, a larger sample size is required for clinical verification, including different tumors. Taken together, we presented an overall picture of lincRNA alterations in cisplatin-resistant progression, and explored the mechanism associated with H19 involvement in this process, which offers new insight into H19 function in ovarian cancer chemotherapy resistance and explores new methods for improving the efficiency of cancer chemotherapy.

## Methods

### Cell Culture and Establishment of Cisplatin Resistant Subline

All cells were maintained in DMEM/F12 (1:1) media supplemented with 10% (v/v) fetal bovine serum. A monoclonal strain was separated by dilution culture method. A2780-DR was derived by incubation with stepwise increasing cisplatin concentrations (from 10 to 20 μmol). After each treatment, the survival cells re-expanded and conventional propagated 4 generation in cisplatin free media. The relative cisplatin resistance was determined by clonogenic assay.

### Clonogenic Assay

Cells were seeded in a 24-well dish at 100 cells/well and allowed to adhere for 24 hours in an incubator after which the cisplatin (5, 10, 20 μmol) (sigma, USA) was added. Cells then replaced with DMEM/F12 (1:1) media after 24 hours incubation. Colonies were manually counted by inverted microscope (Olympus) after 7 days and reported as percent of control. Each individual experiment was performed in triplicate and repeated three times.

### RNA Extraction Preparation for Next-Generation Sequencing

Total RNA was extracted using MiRNeasy Mini kit (Qiagen, USA). Ribosomal RNA was depleted using the RiboMinus™ Eukaryote Kit (Life technologies, USA). Following purification, the poly(A)^−^ or poly(A)^+^ RNA fractions is fragmented into small pieces using divalent cations under elevated temperature. Then the cleaved RNA fragments were reverse-transcribed to construct cDNA library using random primer and connection with the sequencing adapter. Then, the cDNA was amplified by PCR and separated by gel electrophoresis. The average insert size for the paired-end libraries was 300 bp (±50 bp). RNA libraries were then sequenced on the Illumina HiSeq 2000 platform using 100 bp paired-end reads.

### RNA-Sequencing Data Analysis

All RNA-seq data were aligned to hg19 using TopHat/bowtie with default parameters^34^. The mapped reads were assembled using Cufflinks^54^. Transcript abundances were estimated by Cufflinks in Fragments per Kilobase per Million mapped reads (FPKM) for paired-end reads^55^. Only transcripts with length >200nt were retained. In all differential expression tests, a gene was considered significant if the fold change was more than 2 and q value was less than 0.05.

### Patient and Sample Data

41 specimens of HGSC tissues and 13 normal tissues were supplied by Zhejiang Cancer Hospital Biospecimen Repository. The study was approved by the Zhejiang Cancer Hospital ethics committee (zjzlyy 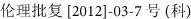) and informed consent was obtained from all subjects. Histopathology and tumor grade were determined by pathology. Stage was determined by a gynecologic oncologist using criteria consistent with FIGO classification. All the methods were carried out in accordance with the approved guidelines by Zhejiang Cancer Research Institution and the patients’ characteristics are detailed in support data (S16).

### RNA Extraction, cDNA Synthesis and qRT-PCR Analysis

Total RNA was extracted using NucleoSpin^®^ TriPrep kit (MACHEREY-NAGEL, Germany). 1 ug of total RNA was used for cDNA synthesis by PrimeScript™ RT reagent Kit (TaKaRa, China). Amplification and melt curve analysis were performed using an ABI 7500 PCR system (Applied Biosystems). Reactions were carried out in a total volume of 20 μl, using SYBR^®^ Premix Ex Taq kit (TaKaRa, China). All PCR reactions were done in triplicates. To confirm the specificity and accuracy of the PCR reaction, PCR products were electrophoresed on a 2% agarose gel and sequenced (Shanghai GeneCore BioTechnologies Co, Ltd. China). GAPDH or ACTB were used as references for lincRNAs. U6 was used as a reference for miRNAs. Relative expression was analyzed using the 2^−ΔΔCt^ method. For patient specimen analysis, eight cases of cDNA hybrid, packed and stored in the low temperature refrigerator. Each PCR detection using one pre mixed reference template. For expression analysis, the experiment was designed to use the reference template as the control, so the relative quantification of gene in test tissue was calculated using the equation: amount of target = 2^−ΔΔCt^, ΔΔCt = (Ctgene–CtACTB) test- (Ctgene–CtACTB) reference. Primer sequences for qPCR are listed in support data (S17).

### Mutation Analysis

DNA was extracted using NucleoSpin TriPrep kit. H19 mutations were analyzed from promoter to 3′ UTR including 5 exons and 4 introns. First, H19 through full length amplification then segmented amplification. PCR products were sequenced by sanger sequencing (Shanghai Sunny Biotechnology). Sequence chromatograms were compared with NCBI reference sequences (GenBank: NG_016165) by MEGA software (Version 5.2.2). Primer sequences for H19 mutation analysis are listed in support data (S18).

### Detection of H19 Expression Profile in Different Time under Cisplatin Treatment

The experiment was performed using CellAmpTM Kit (TaKaRa, China). Cells were cultured in 96-well plate and washed by 125 μl washing buffer once after cell density reached to 80%. 49 μl processing buffer and 1 μl DNase I were added to each well, then incubated for 5 minutes at room temperature. The cell lysate was transferred to microcentrifuge tube and incubated for 5 minutes at 75 °C. qRT-PCR was performed using One Step SYBR^®^ PrimeScript^TM^ RT-PCR Kit (TaKaRa, China) with ABI 7500 System.

### Lentivirus-Mediated Gene Silencing

The shRNA against H19 was designed by the Invitrogen RNAi design tool (http://www.invitrogen.com) and synthesized by Invitrogen, LTD (S19). Non-targeting negative control of shRNA (control) was also synthesized. The oligonucleotides were annealed and inserted into the pLKO.1 siRNA expression vector to generate shRNA. The shRNA against Nrf2 was purchased from Genechem (S19). The cells were plated the day before transfection and allowed to grow to 70–80% confluence. The cells were transiently transfected with H19-shRNA-pLKO.1 or control- pLKO.1 with GBfectene-Elite (Suzhou Genebank Biosciences Inc) in DMEM/F12. The effectiveness of shRNA inhibition was evaluated by real time RT-PCR.

For Lentivirus-mediated RNAi, the 293T cells were transfected with H19-shRNA-pLKO.1 and two kinds of auxiliary packaging vector plasmid with polyethylenimine (PEI). Collected the supernatents and infected A2780-DR cells for 5 hrs with polybrene. To obtain the stable cell line, puromycin selection were performed. The efficiency of H19 inhibition was evaluated by real time RT-PCR.

### Nude Mouse Model

The tumorigenic potential of cell lines was assessed based on their ability to form tumors in 5 week-old female NOD SCID (severe combined immunodeficiency) mice at subcutaneous left gluteal injection sites. 5 × 10^6^ cells was injected in each mouse resuspended in 200 μl of ice-cold serum free medium. After the tumors reached certain size, the mice were sacrificed and the tumors were cut into equal pieced and buried into NOD SCID mouse. Once the tumor size reached to about 0.5 cm × 0.5 cm, divided each mouse line into two groups randomly with one as control and one treat with cisplatin. Tumor formation was assessed three times a week for three weeks with cisplatin injection per week (5 mg/kg). Animals were sacrificed before neoplastic masses reached limit points and the tumor weights were also measured after that. All the methods were carried out in accordance with the approved guidelines by Laboratory Animal Research Center of Zhejiang Chinese Medical University and approved by the ethics committee (SYXK(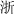)2013–0184).

### Sample Preparation and Label Free Quantitative Proteomic Analysis

Cells were lysed using lysis buffer (4% SDC in 0.1M Tris-Hcl, ph = 8.0) with SIGMAFAST™ Protease Inhibitor (Sigma, USA). Equal amount of proteins from four cell sublines were reduced with 10 mM DTT and alkylated with 25 mM iodoacetamide. In solution digestion was then carried out with sequencing grade modified trypsin/Lys-C (Promega, USA) at 37 °C overnight. The peptides were acidated with 0.5–1% trifluoroacetic acid (TFA) in the final concentration. The SDC was removed by high speed centrifugation. Tryptic peptides were desalted and centrifuged in a speedvac to dry. Then, Tryptic peptides were redissolved in 0.1% FA.

For LC-MS/MS analysis, the peptides were separated by a 90 min gradient elution at a flow rate 0.20 μl/min with a Thermo Scientific EASY-nLC 1000 HPLC system, which was directly interfaced with a Thermo Scientific Q Exactive mass spectrometer. The analytical column was a Thermo Scientific AcclaimR PepMap RSLC column (50 μm ID, 15 cm length, C18, 2 μm, 100 Å). The precolumn was a Thermo Scientific AcclaimR PepMap100 column (100 μm ID, 2 cm length, C18, 5 μm, 100 Å). Mobile phase A consisted of 0.1% formic acid, and mobile phase B consisted of acetonitrile with 0.1% formic acid. The Q Exactive mass spectrometer was operated in the data-dependent acquisition mode using Xcalibur 2.2 SP1 software and there was a single full-scan mass spectrum in the orbitrap (350–2000 m/z, 70,000 resolution) followed by 15 data-dependent MS/MS scans at 27% normalized collision energy (HCD). The MS/MS spectra from each LC-MS/MS run were searched against the “human.fasta” from UniProt (release date of March 19, 2014;68406 entries) using Proteome Discoverer software (Version PD1.4, Thermo Scientific, USA). The search criteria were as follows: full tryptic specificity was required; two missed cleavage was allowed; carbamidomethylation (C) were set as the fixed modifications; the oxidation (M) was set as the dynamic modification; precursor mass tolerances were set at 10 ppm; and the fragment mass tolerance was set at 0.02 Da. The peptide false discovery rate (FDR) was calculated using Percolator provided by PD. Relative protein quantification was performed using MaxQuant software (Version 1.4.0.8). Quantitation was carried out only for proteins with 2 or more unique peptide matches and PEP <0.001. Differently expressed proteins were further confirmed by western blotting.

### Western Blotting

Validation of different expression proteins (NQO1, ALDH1A1, GSR, CDK4 and G6PD, GCLC, GCLM, GSTP1, NRF2) in different cell lines was processed on a traditional way. Briefly, separated denatured proteins on a SDS-PAGE and transferred onto a PVDF membrane, Incubated with primary antibody (1000 × diluted) and secondary antibody (2000 × diluted) sequentially after blocking. The membrane was further washed with PBST and developed using ECL reagents (Pierce, USA). GAPDH was detected as control. The primary antibody of NQO1, ALDH1A1, GSR, CDK4 and G6PD, GCLC, GCLM, GSTP1was purchased for Ptglab, and the NRF2 antibody was purchased from Abcam.

### GSH Detection

Intracellular GSH level was determined by the HPLC method as previously described^56^. Brieftly, Ice-cold sulfosalicylic acid aquous solution (3%, w/w) was added to the collected cells. The mixture was sonicated and the supernatant was vortex-mixed with a solution containing sodium phosphate buffer, DTNB and internal standard p-aminobenzoic acid. The sample was stand at room temperature for 10 min followed by addition of HCl. 10 μl of the supernatant was subjected to HPLC analysis. The HPLC conditions employed a Hypersil GOLD column (100 mm × 2.1 mm i.d., 1.9 μm) (Thermo Scientific), solvent A [0.1% (v/v) TFA] and solvent B [acetonitrile with 0.1% (v/v) TFA]. Solvent B was first increased from 5% to 80% in 13 min, and held at 80% for an additional 3 min. All flow rates were 0.2 ml/min. 326 nm was employed as the detection wavelength. GSH was quantified by detection of the GSH-TNB peak via HPLC.

### Statistical Analysis

Nonparametric test was used to analyze the associations between clinicopathological parameters and lincRNA expression level. The criterion for statistical significance was set at *P* < 0.05 and all calculations were done with SPSS 13.0 software.

### Data availability

The mass spectrometry proteomics data have been deposited to the ProteomeXchange Consortium (http://proteomecentral.proteomexchange.org) via the PRIDE partner repository with the dataset identifier PXD003252 (Username: reviewer85292@ebi.ac.uk; Password: a9PtRbIJ). The RNA-seq data have been deposited to the NCBI SRA database (http://www.ncbi.nlm.nih.gov/Traces/sra/) with the dataset identifier SRP066891.

## Additional Information

**How to cite this article**: Zheng, Z.-G. *et al.* The Essential Role of H19 Contributing to Cisplatin Resistance by Regulating Glutathione Metabolism in High-Grade Serous Ovarian Cancer. *Sci. Rep.* **6**, 26093; doi: 10.1038/srep26093 (2016).

## Supplementary Material

Supplementary Information

Supplementary Dataset 1

Supplementary Dataset 2

Supplementary Dataset 3

Supplementary Dataset 4

Supplementary Dataset 5

Supplementary Dataset 6

Supplementary Dataset 7

Supplementary Dataset 8

Supplementary Dataset 9

Supplementary Dataset 10

Supplementary Dataset 11

Supplementary Dataset 12

Supplementary Dataset 13

Supplementary Dataset 14

Supplementary Dataset 15

Supplementary Dataset 16

Supplementary Dataset 17

Supplementary Dataset 18

Supplementary Dataset 19
