# Supplementary material for: The Essential Role of H19 Contributing to Cisplatin Resistance by Regulating Glutathione Metabolism in High-Grade Serous Ovarian Cancer
Source: Sci Rep. 2016 May 19;6:26093. doi: 10.1038/srep26093 (PMC4872133; doi:10.1038/srep26093)
Supplement: Supplementary Information [file srep26093-s1.doc]

The Essential Role of H19 Contributing to Cisplatin Resistance by Regulating Glutathione Metabolism in High-Grade Serous Ovarian Cancer

Zhi-Guo Zheng^1,2,#^, Hong Xu^1,#, *^, Sha-Sha Suo^1^, Xiao-Li Xu^1^, Mao-Wei Ni^2^, Lin-Hui Gu^2^, Wei Chen^2^, Liang-Yan Wang^1^, Ye Zhao^1^, Bing Tian^1^, Yue-Jin Hua^1,*^

**
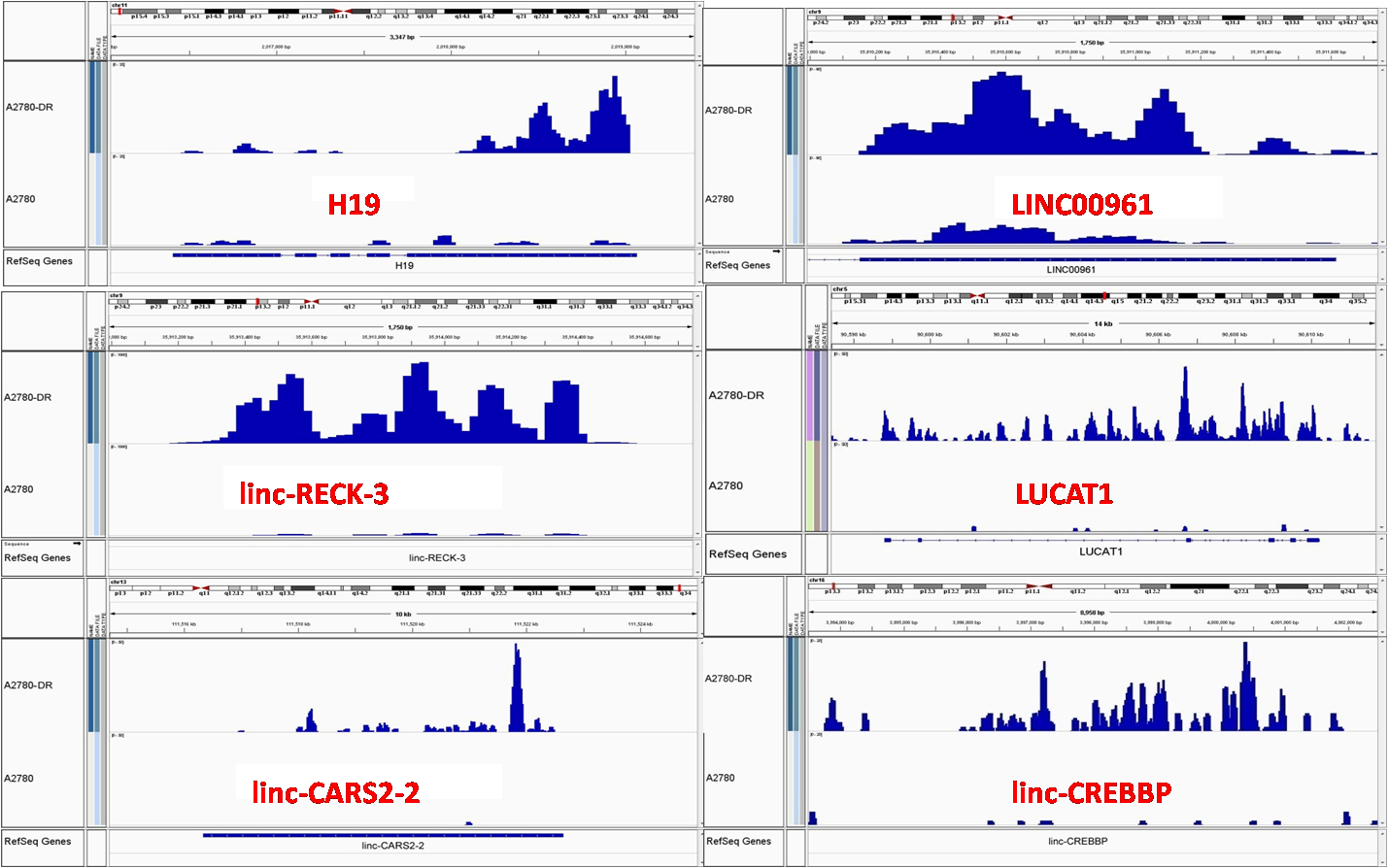
**

**Supplementary Figure 1.** H19, linc-RECK-3, linc-CARS2-2, LINC00961, LUCAT1 and linc-CREBBP expression level in cisplatin sensitive (A2780) and resistant cells (A2780-DR) based on the Integrative Genomics Viewer tool.

**
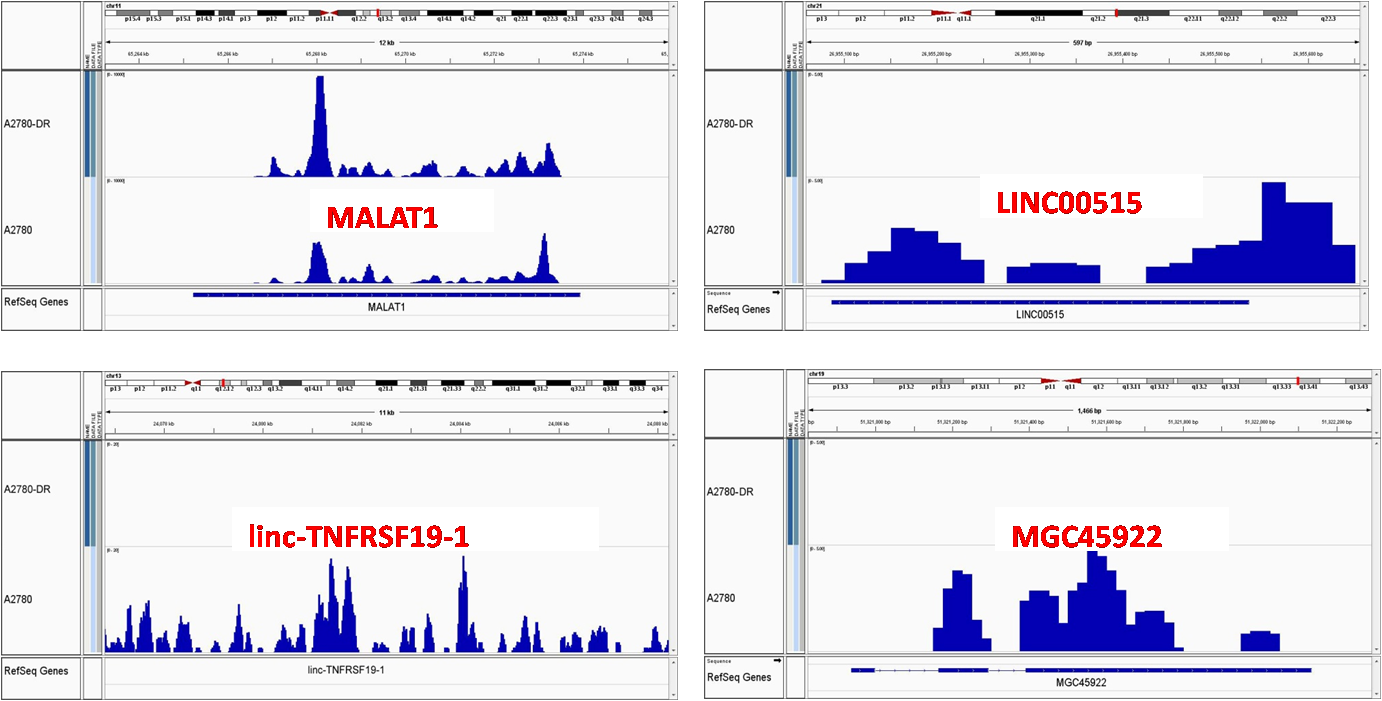
**

**Supplementary Figure 2.** MALAT1, linc-TNFRSF19-1, LINC00515 and MGC45922 expression level in cisplatin sensitive (A2780) and resistant cells (A2780-DR) based on the Integrative Genomics Viewer tool.

**
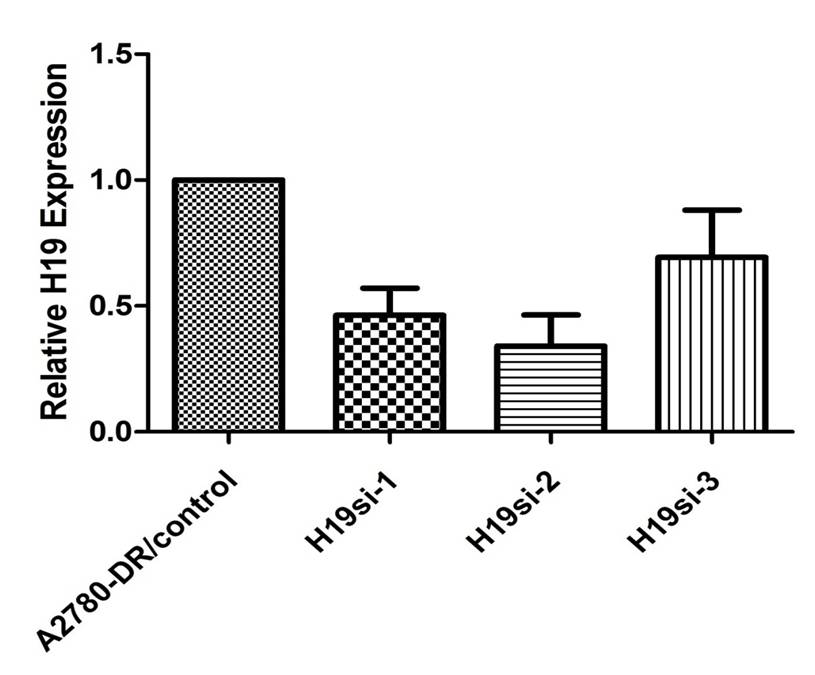
**

**Supplementary Figure 3.** H19 expression level after H19 siRNA interference.

**
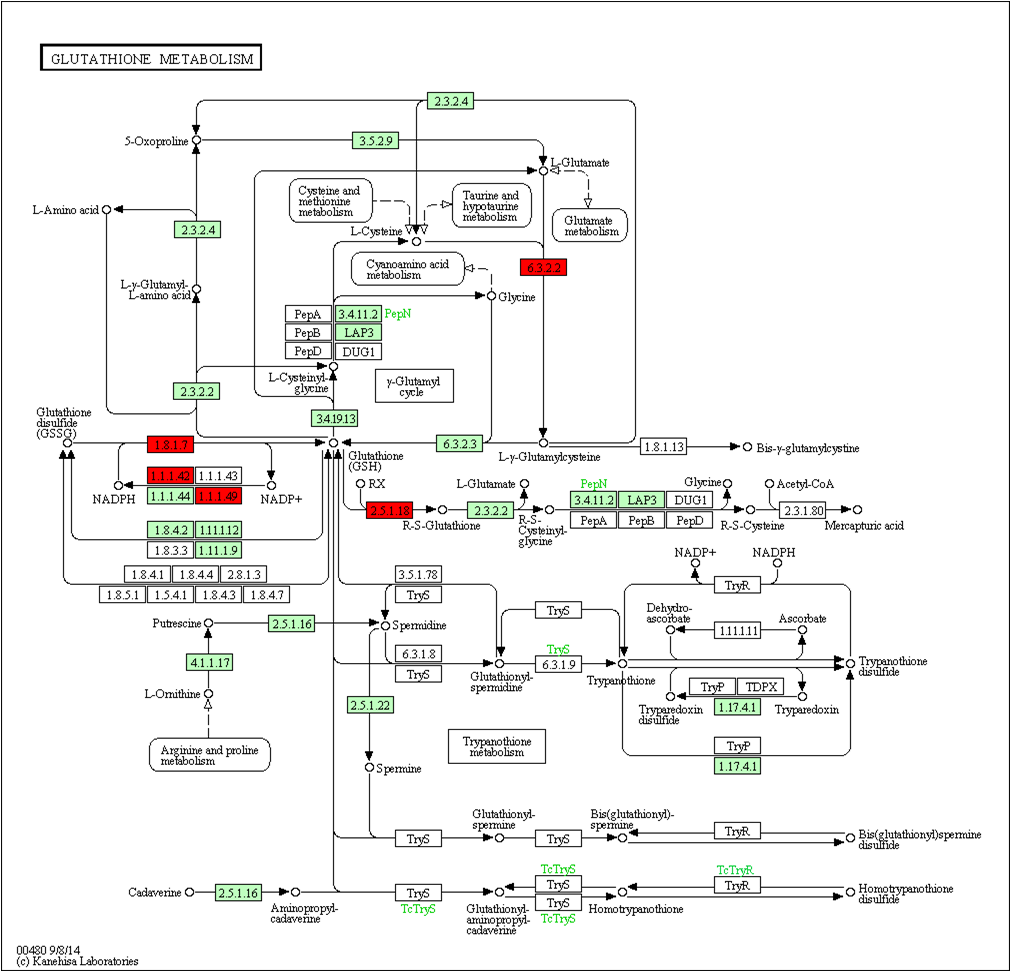
**

**Supplementary Figure 4.** Glutathione metabolism map from KEGG analysis. Six glutathione metabolism proteins showed in red identified by proteomic analysis. 6.3.2.2: GCLC, GCLM; 1.8.1.7: GSR; 1.1.1.42: IDH1; 1.1.1.49: G6PD; 2.5.1.18: GSTP1.


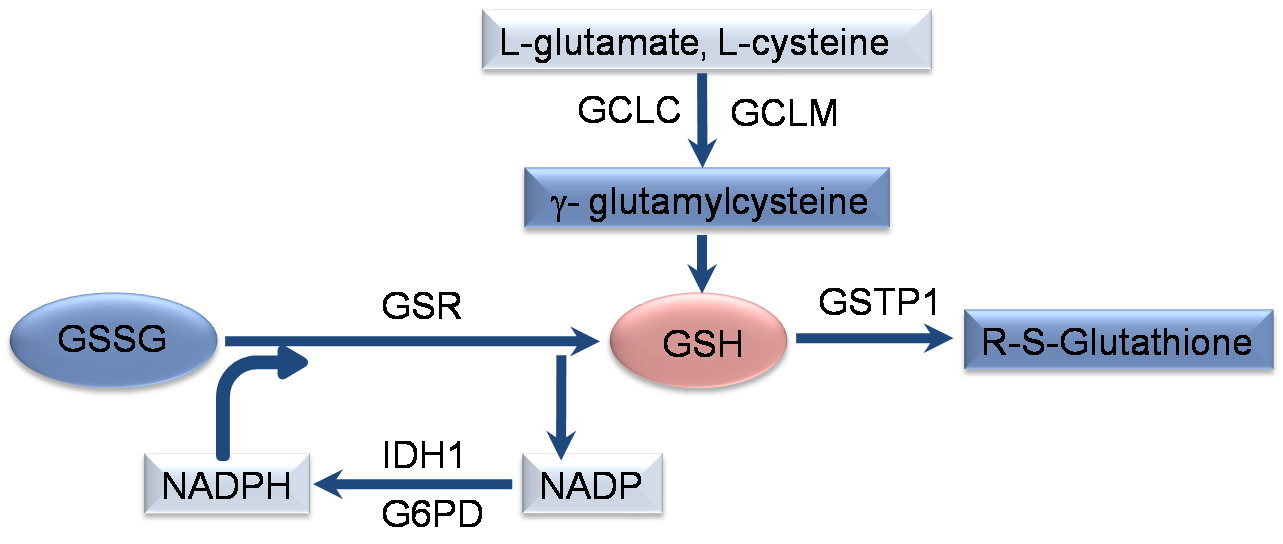


**Supplementary Figure 5.**GSH metabolism genes (GCLC, GCLM, GSR, GSTP1, IDH1) identified by proteomic analysis in this study.


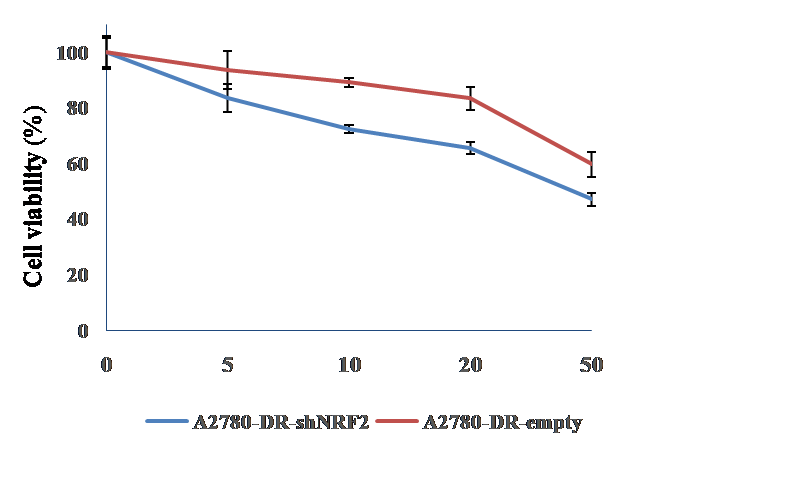


**Supplementary Figure 6.** Cisplatin sensitivity of A2780-DR after NRF2 knockdown. Cisplatin resistant cells (A2780-DR) were transfected with NRF2 interference plasmid (GV248-shNRF2), cells transfected with empty vector was used as negative control.


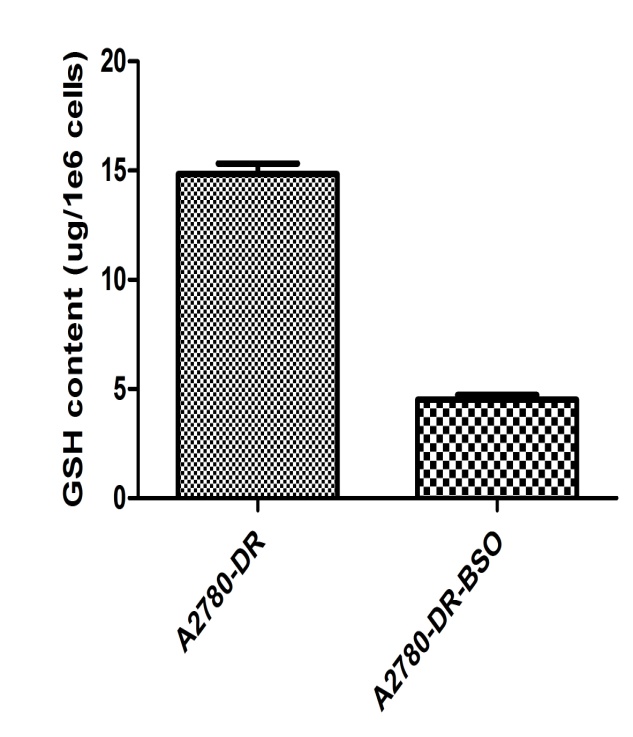


**Supplementary Figure 7.** Intracellular GSH level after BSO treatment. GSH content was measured after cell treated with BSO (100μM) for 24hours.
